# Prognostic superiority of International Prognostic Index over [^18^F]FDG PET/CT volumetric parameters in post-transplant lymphoproliferative disorder

**DOI:** 10.1186/s13550-021-00769-8

**Published:** 2021-03-18

**Authors:** F. Montes de Jesus, D. Dierickx, V. Vergote, W. Noordzij, R. A. J. O. Dierckx, C. M. Deroose, A. W. J. M. Glaudemans, O. Gheysens, T. C. Kwee

**Affiliations:** 1 Department of Nuclear Medicine and Molecular Imaging, University of Groningen, University Medical Center Groningen, Hanzeplein 1, 9700 RB Groningen, The Netherlands; 2 Department of Radiology, University of Groningen, University Medical Center Groningen, Groningen, The Netherlands; 3 Department of Nuclear Medicine, University Hospitals Leuven, Leuven, Belgium; 4 Department of Hematology, University Hospitals Leuven, Leuven, Belgium; 5 Department of Nuclear Medicine, Cliniques Universitaires Saint-Luc, Brussels, Belgium

**Keywords:** Post-transplant lymphoproliferative disorder, 2-[^18^F]fluoro-2-deoxy-d-glucose positron emission tomography, Volumetric parameters, Metabolic tumor volume, Total lesion glycolysis, Prognosis

## Abstract

**Background:**

Post-transplant lymphoproliferative disorders (PTLDs) are a spectrum of hematological malignancies occurring after solid organ and hematopoietic stem cell transplantation. [^18^F]FDG PET/CT is routinely performed at PTLD diagnosis, allowing for both staging of the disease and quantification of volumetric parameters, such as whole-body metabolic tumor volume (MTV) and total lesion glycolysis (TLG). In this retrospective study, we aimed to determine the prognostic value of MTV and TLG in PTLD patients, together with other variables of interest, such as the International Prognostic Index (IPI), organ transplant type, EBV tumor status, time after transplant, albumin levels and PTLD morphology.

**Results:**

A total of 88 patients were included. The 1-, 3-, 5- year overall survival rates were 67%, 58% and 43% respectively. Multivariable analysis indicated that a high IPI (HR: 1.56, 95% CI: 1.13–2.16) and an EBV-negative tumor (HR: 2.71, 95% CI: 1.38–5.32) were associated with poor overall survival. Patients with a kidney transplant had a longer overall survival than any other organ recipients (HR: 0.38 95% CI: 0.16–0.89). IPI was found to be the best predicting parameter of overall survival in our cohort. Whole-body MTV, TLG, time after transplant, hypoalbuminemia and PTLD morphology were not associated with overall survival.

**Conclusion:**

[^18^F]FDG PET/CT whole-body volumetric quantitative parameters were not predictive of overall survival in PTLD. In our cohort, high IPI and an EBV-negative tumor were found to predictors of worse overall survival while kidney transplant patients had a longer overall survival compared to other organ transplant recipients

## Background

Post-transplant lymphoproliferative disorders (PTLDs) are a spectrum of hematological malignancies occurring after solid organ and hematopoietic stem cell transplantation in the setting of pharmacological immunosuppression. In this already vulnerable population, PTLD constitutes a serious health burden, associated with high morbidity and mortality [1]. Although risk-stratified sequential treatment and the introduction of Rituximab have improved outcome, reported 3-year overall survival remains low, ranging from 40 to 70% [2–6]. In an attempt to stratify high-risk patients, various prognostic makers and different prognostic scores have been suggested.

Several classical lymphoma-specific markers have been identified as consistent predictors of overall survival in PTLD cohorts. Indeed, multiple studies have identified age, performance status, elevated lactate dehydrogenase and extra-nodal disease as independent predictors of overall survival [2, 3, 7–11]. Additionally, several other markers have been reported to be predictive of survival including: number/location of involved sites, morphological subtype, time from transplantation, presence of B-symptoms, albumin levels, serum creatine, gender and organ transplanted [2, 3, 7–10, 12]. Different prognostic scores have also been shown to be significant predictors of overall survival [2, 3, 5, 7–9, 12]. The International Prognostic Index (IPI) is a clinically validated tool in the prognostication of aggressive non-Hodgkin lymphoma and its value in PTLD has been established in the PTLD-1 trial [13, 14]. Taking into consideration the particularities of post-transplantation immunocompromised patients, Caillard and colleagues have proposed an PTLD specific prognostic score after kidney transplantation, which nevertheless does not seem to surpass the performance of the IPI [12, 15]. Although current prognostic models allow for some degree of stratification, they fail to perform consistently across all cohorts and are seldomly employed clinically. Therefore, there is a need for new clinically applicable markers.

Quantification of whole-body tumor metabolism may provide additional information, not perceptible with current clinical and biological markers. 2-[^18^F]fluoro-2-deoxy-D-glucose positron emission tomography/computed tomography ([^18^F]FDG PET/CT) not only allows for anatomical lesion localization, but also for quantification of volumetric parameters, such as whole-body metabolic tumor volume (MTV) and total lesion glycolysis (TLG). As [^18^F]FDG PET/CT is considered standard-of-care in many institutions and current commercial software packages allow for semi-automatic metabolic quantification, MTV and TLG may become clinically feasible prognostic tools [16]. In immunocompetent lymphoma patients and in particular diffuse large B-cell lymphoma (DLBCL), high baseline MTV and TLG have been reported to be associated with worse survival [17]. However, these volumetric parameters have yet to be evaluated in PTLD.

We performed a retrospective study to determine the prognostic value of baseline whole-body MTV and TLG measurements in patients with newly diagnosed, biopsy-proven PTLD as a primary research goal. Prognostic value of IPI and other markers of interest were analyzed as secondary outcome parameters.

## Methods

### Study design and patient selection

This retrospective study was performed at the University Medical Center Groningen (UMCG) and the University Hospitals Leuven (UZ Leuven) including biopsy-proven de novo PTLD patients between 2009 and 2019. Patients included in this study underwent an [^18^F]FDG PET/CT at baseline with reconstruction parameters according to The European Association of Nuclear Medicine Research Ltd (EARL) recommendations [18, 19]. Patients excluded were those in whom accurate segmentation either semi-automatically or visually was not possible (i.e., areas of high background physiological uptake), previously treated PTLD or those with more than 30 days between histopathological confirmation and the [^18^F]FDG PET/CT. The study was conducted in accordance with the ethical principles of the Declaration of Helsinki and with the approval of the respective ethical committees.

[^18^F]FDG PET/CT acquisition and semi-quantification.

[^18^F]FDG PET/CT scans were performed using a Siemens Biograph mCT 40- or 64-slice (Siemens Healthcare, Knoxville, Tennessee, United States) at the UMCG and a Siemens Biograph 16 HiRez, Siemens Truepoint 40 (Siemens Healthcare, Erlangen, Germany) or GE Healthcare Discovery MI4 (GE Healthcare, Chicago, IL, USA) at the UZ Leuven. Patients fasted for a minimum of 6 h and glucose levels were targeted at < 11 mmol/L (range: 3.3 to 14.5 mmol/L) before intravenous [^18^F]FDG administration (range: 3 to 4.25 MBq [^18^F]FDG/kg body weight). Sixty minutes after [^18^F]FDG administration a low-dose CT scan was performed, immediately followed by a whole-body (vertex to mid-thigh) PET scan using a multi-bed position, with 70 to 180 s per bed position. Low-dose CT data were used for attenuation correction of the PET images.

Semi-quantification of volumetric parameters was performed on the Hermes Hybrid 3D software (Hermes Medical Solutions AB, Stockholm, Sweden) by F.M.J. (nuclear medicine research fellow) blinded for all other results with the support of two experienced nuclear physicians (A.W.J.M.G. & W.N.). Extracted volumetric parameters included: whole-body MTV defined as, the total metabolically active volume of the segmented tumors, and whole-body TLG, defined as whole-body MTV × mean standardized uptake value (SUV_mean_) contained within the volume of interest. TLG was corrected for fasting glucose using the formula: (TLG × fasting glucose in mmol/L)/5. MTV and TLG were interpreted as continuous variables. Lesion segmentation was performed with the “Tumor Finder” application in Hermes Hybrid 3D, in line with PERCIST recommendations [20]. Based on a 14.1-ml spherical volume placed in the right lobe of the liver, lesions above a threshold of 1.5 × liver SUV_mean_ + 2 standard deviations were selected. If the use of the right lobe of the liver as a reference region was not possible (ongoing liver pathology which would impact physiological liver metabolism i.e. diffuse metastatic disease), a 1.6-ml spherical volume was placed in the mediastinal blood pool and lesions selected based on a threshold above 2 × mediastinal blood pool SUV_mean_ + 2 standard deviations [20]. Lesions not automatically segmented but suspected of malignancy were manually added, while any metabolically active focus interpreted as physiological was removed. During manual segmentation, particular attention was paid to extra-nodal lesions and splenic involvement. By diffuse splenic involvement the whole spleen was segmented while by focal involvement, lesions with [^18^F]FDG uptake higher than background were selected.

International Prognostic Index and prognostic parameters.

The IPI score of each patient was calculated retrospectively, interpreted as a continuous variable [13]. Other potential prognostic markers evaluated were: organ transplant type, Epstein-Barr virus (EBV) tumor status by in situ hybridization (EBV-positive *versus* EBV-negative), time after transplant (early-PTLD ≤ 1 year *versus* late-PTLD > 1 year), hypoalbuminemia (defined as albumin < 35 g/L) and PTLD morphology (non-destructive PTLD, polymorphic PTLD, monomorphic PTLD or classic Hodgkin lymphoma-type PTLD).

### Statistical analysis

Categorical variables were presented as counts and percentages, while continuous variables as median with interquartile range (IQR). Variables were graphically checked for normality. Cox proportional hazards model was used for survival analysis with overall survival as endpoint, defined as time from diagnosis until death (from any cause). Surviving patients were censored at the last date of follow-up as mentioned in the patient record files. A combination of backward and forward likelihood-ratio model was used, with probability for stepwise removal set at p ≤ 0.1 and probability for stepwise entry set at p ≤ 0.05. Variables remaining in the backward likelihood-ratio model were further analyzed with a forward likelihood-ratio model and dummy variables created for categorical variables. The stability of the model selection procedure was tested by bootstrap resampling with 1000 replications and statistical significance set at p ≤ 0.05. Results were reported as hazard ratio (HR), with a 95% confidence interval (95% CI). Log base 10 transformation was used for highly skewed variables. Correlations between the variables included in the model were assessed using Spearman’s rank correlation coefficient (ρ). Correlations were categorized as very weak (ρ = 0–0.19), weak (ρ = 0.20–0.39), moderate (ρ = 0.40–0.5), strong (ρ = 0.60–0.79) and very strong (ρ = 0.80–1.00). The following list of variables were considered in the model: MTV, TLG, IPI, organ transplant type, EBV tumor status, time after transplant, albumin levels and PTLD morphology. Statistical and graphical analysis were performed using SPSS, version 23.0 (IBM Corporation, Armonk, NY, USA).

## Results

### Demographic characteristics

A total of 116 PTLD patients with baseline, EARL reconstructed [^18^F]FDG PET/CT were identified from the patient record files. From these patients, 13 were excluded because accurate segmentation was not possible (mostly due to central nervous system-PTLD). Seven patients were excluded due to previously treated PTLD and in 5 patients, histopathological confirmation was not available within 30 days of the [^18^F]FDG PET/CT. Finally, 2 patients were excluded because fasting glucose prior to the [^18^F]FDG PET/CT scan was not reported and in 1 patient multiple variables could not be retrieved, preventing inclusion in the survival model. In total, 88 patients were included in this study, 47 patients from UZ Leuven and 41 from the UMCG. There were 53 (60%) males and 35 (40%) females with a median age at diagnosis of 51 years (IQR: 33.3–62.8 years). Kidney was the most often transplanted organ in 35% of patients, followed by lung (23%) and liver (17%). Morphology was predominantly monomorphic (77%), with 57% of all tumors being EBV-positive. The majority of cases (76%) occurred more than 1-year post transplantation, defined as late-PTLD. Median baseline IPI was 2 (IQR: 1–3). Baseline therapy was most often given as single-agent Rituximab (66%) or chemotherapy (21%). Forty-one percent of patients were deceased mostly due to PTLD (53%) or therapy-related complications (17%). Median whole-body MTV and TLG values were 272 (IQR:42–566) and 1825 (IQR: 232–5610), respectively (Table 1).

**Table 1:** Demographic characteristics n = 88

Gender	
Male	n = 53 (60%)
Female	n = 35 (40%)
Age (years)	
Median	51
IQR	33–63
Organ transplanted	
Kidney	n = 31 (35.2%)
Lung	n = 20 (22.7%)
Liver	n = 15 (17.1%)
Hematopoietic stem cell transplantation	n = 10 (11.4%)
Heart	n = 6 (6.8%)
Multi-organ	n = 6 (6.8%)
Morphology	
Non-destructive	n = 8 (9%)
Polymorphic	n = 10 (11.4%)
Monomorphic	n = 68 (77.3%)
Classic Hodgkin Lymphoma	n = 2 (2.3%)
EBV tumor status	
Positive	n = 50 (56.8%)
Negative	n = 38 (43.2%)
Onset PTLD	
Early (< 1 year)	n = 21 (23.9%)
Late (> = 1 year)	n = 67 (76.1%)
Ann Arbor staging	
I	n = 7 (8%)
II	n = 12 (13.6%)
III	n = 12 (13.6%)
IV	n = 57 (64.8%)
Extranodal involvement	
Yes	62 (70.5%)
No	26 (29.5%)
Hypoalbuminemia	
Yes	n = 35 (40%)
No	n = 53 (60%)
International Prognostic Index	
0	n = 9 (10.2%)
1	n = 13 (14.8%)
2	n = 24 (27.3%)
3	n = 32 (36.3%)
4	n = 8 (9.1%)
5	n = 2 (2.3%)
Baseline therapy	
Rituximab	n = 58 (65.9%)
Chemotherapy	n = 19 (21.6%)
Other	n = 4 (4.5%)
Missing	n = 7 (8%)
Outcome*	
Alive	n = 51 (58%)
Deceased	n = 36 (40.9%)
Lost to follow-up	n = 1 (1.1%)
Cause of Death	
PTLD	n = 19 (53%)
Therapy-related complication	n = 6 (17%)
Other/Unknown	n = 11 (30%)
Metabolic tumor volume (mL)	
Median	272
IQR	42–566
Total lesion glycolysis (grams)	
Median	1825
IQR	232–5610

*Median follow-up: Alive—58 months (IQR: 35–101); Deceased—5 months (IQR: 2–9)

### Survival analysis

**Fig. 1:**
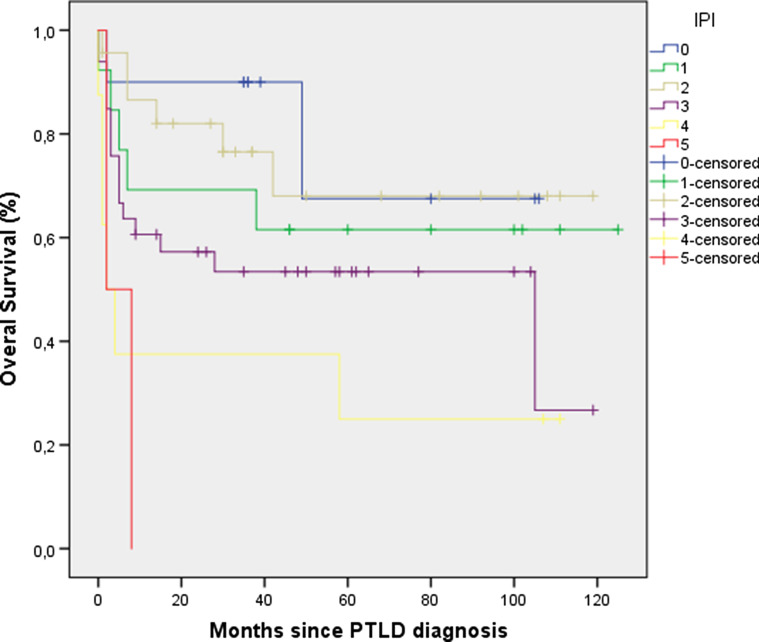
Overall survival International Prognostic Index

**Fig. 2:**
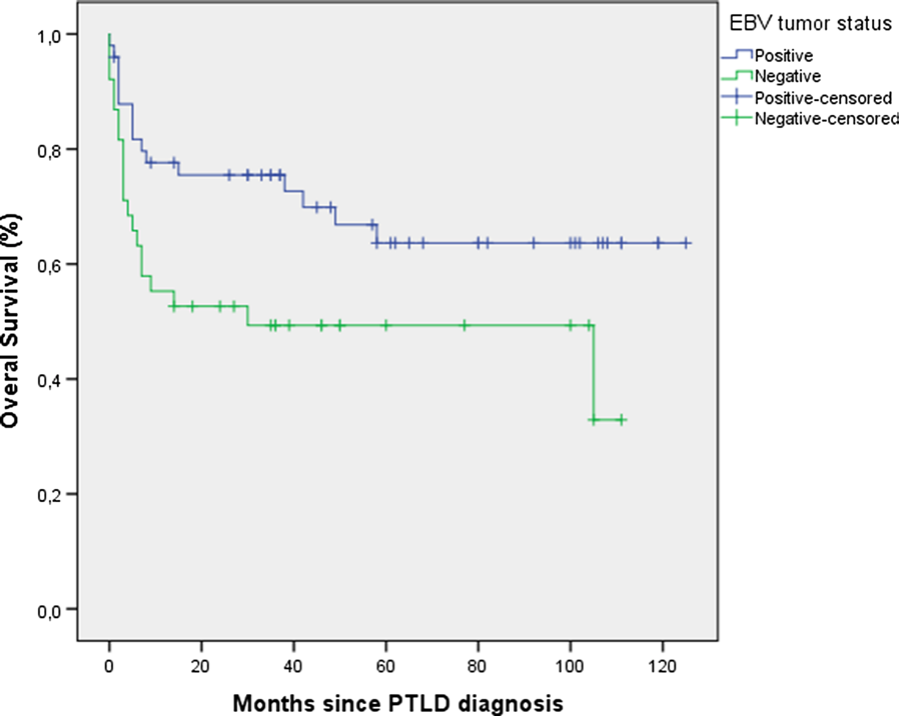
Overall survival EBV tumor status

The 1-, 3-, 5-year overall survival rates were 67%, 58% and 43% respectively. Median survival for all patients was 35 months (IQR: 5–67), with a median follow-up for the 51 living patients of 58 months (IQR: 35–101). MTV and TLG underwent log-transformation due to the right-sided skewed distribution. In backwards stepwise elimination, TLG and MTV were eliminated in step 3 and step 4, respectively and were not included in further analysis. Glucose uncorrected TLG values were likewise not prognostic of overall survival (data not shown). IPI (p = 0.01), EBV status of the tumor (p = 0.01) and transplanted organ (p = 0.04) were retained in the model (Table 2). These variables were selected for forward selection analysis and the categorical ‘transplanted organ’ variable coded into a dummy variable for each organ transplant type (kidney, lung, liver, HSCT, heart or multiorgan). A high IPI (HR: 1.56, 95% CI: 1.13–2.16) and an EBV-negative tumor (HR: 2.71, 95% CI: 1.38–5.32) were associated with lower overall survival (Figs. 1, 2). Patients with a kidney transplant had longer overall survival than transplant recipients of any other organ (HR: 0.38 95% CI: 0.16–0.89). IPI was the first variable to be included in the forward selection model, suggesting it as the best fitting variable in our model. All variables retained statistical significance after bootstrapping (Table 3). Variables included in the final model (IPI, EBV tumor status and transplanted organ-kidney) were not correlated to each other.

**Table 2:** Overall survival analysis—backward stepwise elimination

Backward model elimination		p-value
Variables removed	Step 1	
	PTLD morphology	0.79
	Step 2	
	Onset PTLD	0.9
	Step 3	
	Total lesion glycolysis	0.18
	Step 4	
	Metabolic tumor volume	0.65
	Step 5	
	Hypoalbuminemia	> 0.1
Variables retained	Step 6	
	Transplanted organ	0.04
	EBV tumor status	0.01
	International Prognostic Index	0.01

**Table 3:** Overall survival analysis—forward stepwise selection and Bootstrapping

Forward model selection			Bootstrapping
	HR (95% CI)	p-value	p-value
Step 1			
International Prognostic Index		≤ 0.01	
Step 2			
International Prognostic Index		≤ 0.01	
EBV tumor status		≤ 0.01	
Final model			
Step 3			
International Prognostic Index	1.56 (1.13–2.16)	≤ 0.01	0.01
EBV tumor status		≤ 0.01	0.01
EBV-negative	2.71 (1.38–5.32)		
Transplanted organ		0.02	0.02
Kidney	0.38 (0.16–0.89)		

## Discussion

In this 88-patient PTLD cohort, multivariable overall survival analysis indicated that a high IPI and an EBV-negative tumor were associated with lower overall survival. Kidney transplant patients seemed to have a longer overall survival compared to other transplant organ recipients. Whole-body MTV, TLG, time after transplant, hypoalbuminemia and PTLD morphology were not associated with overall survival. Based on these findings, clinical use of IPI may be applicable in PTLD patients while [^18^F]FDG PET/CT derived volumetric parameters do not to add any prognostic value.

In contrast with other [^18^F]FDG-avid lymphomas in immunocompetent patients (IC-lymphomas), MTV and TLG measurements were not predictive of overall survival in our PTLD cohort. Despite some conflicting results, several studies have reported high baseline MTV and TLG to be associated with worse overall survival in IC-lymphomas [17, 21–24]. Nevertheless, characteristics inherent to PTLD prevent direct extrapolation of these previous findings. PTLD occurs in immunocompromised patients after solid organ/hematopoietic stem cell transplantation with distinct pathophysiology and clinical manifestations [25]. PTLD in EBV-positive cases is more reliant on the oncogenic effects of the virus, with greater infiltration of immune cells such as cytotoxic T-cells and M2 macrophages. On the other hand, lymphoma in immunocompetent patients, is characterized by a greater number of genetic mutations (as compared to EBV-positive cases) [26, 27]. Therefore, it can be speculated that while [^18^F]FDG uptake may be mostly due to underlying inflammation in EBV-positive PTLD, genetic mutations may account for the [^18^F]FDG uptake observed in IC-lymphomas (and in EBV-negative cases). This is particularly true for the p53 mutations, associated with higher SUV uptake [28]. Another distinct feature of PTLD as compared to IC-lymphomas, is the higher incidence of extra-nodal disease, particularly in the allograft [29]. Similar to IC-lymphomas, extra-nodal disease involvement has been associated with lower overall survival in PTLD patients [2, 5, 7]. Yet, as PTLD is characterized by frequent extra-nodal disease, the metabolic tumor volume may be less significant than the location of the lesions in this patient population. In previous studies, involvement of the central nervous system, bone marrow, graft organ and serous membranes have all been associated with poorer survival in PTLD patients [2, 10, 12]. Therefore, even a small tumor with low MTV and TLG may greatly impact survival depending on the extra-nodal lesion location. Finally, considering the recent studies on the prognostic value of baseline whole-body volumetric parameters in IC-lymphomas, the vast majority uses optimal cutoff values derived from retrospective receiver operating curve analysis [17, 30]. Consequently, the prognostic value of whole-body MTV and TLG may have been frequently overestimated in previous studies.

From the remaining parameters evaluated in the multivariable analysis model, high IPI (HR: 1.56, 95% CI: 1.13–2.16) was the first variable to be included in our forward likelihood-ratio model. Although the IPI is widely used for aggressive lymphomas in immunocompetent patients, some authors have questioned its applicability to PTLD. While some studies have demonstrated the prognostic value of IPI in PTLD, others have argued that their own PTLD specific model was superior at predicting survival or that IPI failed to predict survival altogether [3, 7, 8, 10, 31]. Criticism against the use of the IPI in PTLD has included: the inappropriate cutoff age (taking into consideration the vulnerability of this patient population) and the inability of the IPI to account for the predominance of extra-nodal lesions in PTLD patients (leading to generalized higher IPI scores in PTLD as compared to IC-lymphomas) [12]. Similar to the IPI, the role of EBV tumor status on the survival of PTLD patients has been inconsistent. While some studies have found EBV tumor status to be a predictor of overall survival in either univariable or multivariable models, others have dismissed these findings [3, 5, 9, 32]. Although the role of EBV tumor status is undefined, evidence seems to be mounting on the hypothesis that EBV-positive and EBV-negative PTLD are distinct entities [33–35]. EBV-negative PTLD has been shown to have a complex genetic profile with a distinct microenvironment, similar to that found in IC-lymphomas [33, 34]. Furthermore, a recent study by Menter et al. has identified three distinct PTLD subgroups, two of which related to EBV infection status [33]. How this distinction may affect overall survival was not reported but the EBV-negative cluster had a poorer relapse-free survival compared to the other two groups. Considering that EBV tumor status and time of onset after transplant are usually associated, it is perhaps surprising that EBV tumor status was prognostic of survival in our analysis while time of onset after transplant was not. However, in our cohort these two variables were only moderately correlated (ρ = 0.43) which may explain the present results. Finally, kidney transplant patients seemed to have a longer overall survival in our cohort. Although a crucial parameter, specific to PTLD patients and not included in the IPI, few studies have focused on the type of organ transplant. In a study by Dierickx et al., liver transplant patients with PTLD were identified as having a worse overall survival as compared to PTLD patients after kidney transplant [10]. One possible explanation is the higher number of kidney transplants performed per year and the subsequent greater clinical expertise. Another reason may be the ability to better adjust immunosuppression in order to preserve allograft function and to perform dialysis is case of graft failure.

The retrospective nature of this study and the lack of model validation constitute an inherent limitation. Additionally, group distribution was not balanced, with only 8 non-destructive PTLD and 10 polymorphic PTLD cases regarding morphology and only 6 heart and 6 multi-organ transplant patients. As a result, we may not have had enough patients to reach statistical significance in these subgroups. Our cohort also included 3 patients with plasma glucose levels above the 11 mmol/L recommended by the European Association of Nuclear Medicine [36]. Nevertheless, when excluding these patients from our analysis, the overall results did not change. Finally, in the present study we limited our analysis to overall survival as a sole endpoint. This was however deliberately chosen, as other common endpoints such as progression-free survival or disease-free survival may have introduced incorporation or assessment bias into our results.

The lack of established prognostic parameters in PTLD highlights the challenging and complex nature of this disease. Its rarity, broad pathologic spectrum, heterogenous patient population and multiple treatment modalities have difficulted model validation in large patient cohorts. Whole-body MTV and TLG were not applicable for PTLD prognostication. In our cohort and similar to the PTLD-1 trial, IPI may be applicable, but is far from perfect as illustrated by the conflicting results in the literature. Due to the distinct pathophysiology and epidemiology of PTLD, it remains counter intuitive to use IPI instead of a PTLD specific prognostic score. Therefore, future prospective multicenter trials to determine more appropriate prognostic parameters and scores for PTLD are encouraged. Additionally, end-of-treatment [^18^F]FDG PET/CT has been reported to identify PTLD patients with low risk of relapse and volumetric parameters may further be explored in this group [37, 38].

## Conclusion

[^18^F]FDG PET/CT whole-body volumetric quantitative parameters were not predictive of overall survival in PTLD. In our cohort, high IPI and an EBV-negative tumor were found to predictors of worse overall survival while kidney transplant patients had a longer overall survival compared to other organ transplant recipients.

## Data Availability

The datasets used and/or analyzed during the current study are available from the corresponding author on reasonable request.

## Abbreviations

CI: Confidence interval
CT: Computed tomography
DLBCL: Diffuse large B-cell lymphoma
EARL: The European Association of Nuclear Medicine Research
[^18^F]FDG: 2-[^18^F]fluoro-2-deoxy-D-glucose
HSCT: Hematopoietic stem cell transplantation
IPI: International Prognostic Index
IQR: Interquartile range
MTV: Metabolic tumor volume
PET: Positron emission tomography
PTLD: Post-transplant lymphoproliferative disorder
SUV: Standardized uptake value
TLG: Total lesion glycolysis
UMCG: University Medical Center Groningen
UZ Leuven: University Hospital Leuven
